# Emergence of a dalbavancin induced glycopeptide/lipoglycopeptide non-susceptible *Staphylococcus aureus* during treatment of a cardiac device-related endocarditis

**DOI:** 10.1038/s41426-018-0205-z

**Published:** 2018-12-05

**Authors:** Manuel Kussmann, Matthias Karer, Markus Obermueller, Katy Schmidt, Wolfgang Barousch, Doris Moser, Marion Nehr, Michael Ramharter, Wolfgang Poeppl, Athanasios Makristathis, Stefan Winkler, Florian Thalhammer, Heinz Burgmann, Heimo Lagler

**Affiliations:** 1 Department of Medicine I, Division of Infectious Diseases and Tropical Medicine, Medical University of Vienna, Vienna, Austria; 2 Center for Anatomy and Cell Biology, Division of Cell and Developmental Biology, Medical University of Vienna, Vienna, Austria; 3 Department of Laboratory Medicine, Division of Clinical Microbiology, Medical University of Vienna, Vienna, Austria; 4 Department of Cranio-Maxillofacial and Oral Surgery, Medical University of Vienna, Vienna, Austria; 5 Department of Tropical Medicine; Bernhard Nocht Institute for Tropical Medicine & I. Dep. Of Medicine, University Medical Center Hamburg-Eppendorf, Hamburg, Germany; 6 Military Medical Cluster East, Austrian Armed Forces, Vienna, Austria

## Abstract

In the present study, we demonstrated the emergence of dalbavancin non-susceptible and teicoplanin-resistant *Staphylococcus aureus* small colony variants which were selected in vivo through long-term treatment with dalbavancin. A 36-year-old man presented with a cardiac device-related *S. aureus* endocarditis and received long-term therapy with dalbavancin. Consecutively, two glycopeptide/lipoglycopeptide susceptible and two non-susceptible *S. aureus* isolates were obtained from blood cultures and the explanted pacemaker wire. The isolates were characterized by: standard typing methods, antimicrobial susceptibility testing, auxotrophic profiling, proliferation assays, scanning and transmission electron microscopy, as well as whole genome sequencing. The isolated SCVs demonstrated a vancomycin-susceptible but dalbavancin non-susceptible and teicoplanin-resistant phenotype whereof the respective MICs of the last isolate were 16- and 84-fold higher than the susceptible strains. All four strains were indistinguishable or at least closely related by standard typing methods (*spa*, MLST, and PFGE), and whole genome sequencing revealed only eight sequence variants. A consecutive increase in cell wall thickness (up to 2.1-fold), an impaired cell separation with incomplete or multiple cross walls and significantly reduced growth rates were observed in the present study. Therefore, the mutations in *pbp2* and the DHH domain of GdpP were identified as the most probable candidates due to their implication in the biosynthesis and metabolism of the staphylococcal cell wall. For the first time, we demonstrated in vivo induced dalbavancin non-susceptible/teicoplanin resistant, but vancomycin and daptomycin susceptible *S. aureus* SCVs without lipopeptide or glycopeptide pretreatment, thus, indicating the emergence of a novel lipoglycopeptide resistance mechanism.

## Introduction

Dalbavancin, a semisynthetic lipoglycopeptide derived from the teicoplanin-like antibiotic A-40926, is presently approved for single-dose treatment of acute Gram positive bacterial skin and soft tissue infections in adult patients^1,2^. Dalbavancin targets the bacterial cell wall synthesis like first generation glycopeptide antibiotics, but was shown to be more active against *Staphylococcus* spp. in vitro compared to quinupristin/dalfopristin, linezolid, teicoplanin, or vancomycin^3,4^.

With a half-life (t_1/2_) of 181–216 h dalbavancin further exhibits unique pharmacokinetic properties ideal for infections requiring prolonged antimicrobial treatment^5–7^. Thus, dalbavancin has been increasingly used off-label for indications like infective endocarditis, osteomyelitis, or foreign body infections, in which outpatient parenteral antimicrobial therapy (OPAT) is awaited but not practicable. Data on the efficacy of long-term OPAT with dalbavancin, as well as on the risk to select for resistant strains, however, are limited to few animal studies, single case reports or small case series in humans.

To date, in vivo induced lipoglycopeptide resistance was only described in a single *S. aureus* urine isolate after long-term therapy with vancomycin followed by dalbavancin^8^. Genetic alterations in the *yvqF* gene were considered to be the most likely cause based on the previously reported implication in a vancomycin resistance pathway^8,9^. Other frequently identified mutations found in clinical isolates with reduced glycopeptide susceptibility were *graRS*, *rpoB, vraRS*, or *walKR* which are mostly involved in the biosynthesis or metabolism of the staphylococcal cell wall^10–15^.

Likewise, *S. aureus* small colony variants (SCVs) demonstrate altered antimicrobial resistance patterns due to changes in their metabolism (e.g., cell wall biosynthesis, amino acid transport, membrane potential) and are frequently selected by environmental stress such as long-term antimicrobial treatment, challenging both microbiologists and clinicians^16–18^.

In the present study, we consecutively isolated two wild-type strains and two dalbavancin non-susceptible and teicoplanin-resistant SCVs from a patient with a cardiac device-related *S. aureus* infection during a long-term OPAT with dalbavancin. Furthermore, we investigated antimicrobial resistance patterns, auxotrophic profiles, electron microscopic cell morphologies, and the genomic characteristics of these strains to identify potentially underlying mechanisms of the observed in vivo induced increase of dalbavancin MICs.

## Results

### Antimicrobial susceptibility testing

Antimicrobial susceptibility tests were performed with all four isolates (DR-I1–DR-I4) using cefoxitin, ciprofloxacin, clindamycin, dalbavancin, daptomycin, doxycycline, fosfomycin, fusidic acid, gentamicin, linezolid, oxacillin, rifampicin, teicoplanin, trimethoprim/sulfamethoxazole, and vancomycin (Table 1,2). The initially isolated blood culture strain DR-I1 was susceptible to cefoxitin, dalbavancin, teicoplanin, and vancomycin but resistant to fusidic acid. For the second blood culture isolate DR-I2 an increased cefoxitin MIC of 6 mg/L, indicating methicillin resistance, as well as a markedly decreased fusidic acid MIC of 0.064 mg/L were observed. The two SCV isolates obtained during OPAT with dalbavancin (DR-I3 and DR-I4) demonstrated increased oxacillin (2 mg/L and 3 mg/L) and cefoxitin (6 mg/L and 8 mg/L) MICs, as well as a dalbavancin non-susceptible (0.5 mg/L and 1.0 mg/L) phenotype. Furthermore, vancomycin MICs remained at 2 mg/L whereas the teicoplanin MIC increased from 2 mg/L (DR-I3) to 16 mg/L (DR-I4).

**Table 1:** Antimicrobial susceptibility profiles of the four isolated *S. aureus* strains. All MICs were obtained by use of E-tests, except for dalbavancin where a broth microdilution assay was performed additionally

	DR-I1	DR-I2	DR-I3	DR-I4
Oxacillin	0.500	2.000	2.000	3.000
Cefoxitin	4.000	6.000	6.000	8.000
Vancomycin	1.500	1.500	2.000	2.000
Teicoplanin	0.190	0.380	2.000	16.000
Dalbavancin	0.047	0.047	0.190	0.380
Dalbavancin (BMA)^a^	0.063	0.063	0.500	1.000
Daptomycin	0.380	0.380	0.500	0.750
Trimethoprim/Sulfamethoxazole (E-test)	0.064	0.047	0.064	0.064
Fusidic acid	>256.000	0.064	0.250	0.190
Doxycyclin	0.250	0.047	0.190	0.190
Gentamicin	0.500	0.190	0.032	0.064
Clindamycin	0.125	0.047	0.047	0.032
Linezolid	2.000	1.000	2.000	2.000
Ciprofloxacin	0.750	0.500	0.190	0.250
Fosfomycin	1.500	4.000	0.500	0.500
Rifampicin	0.016	0.006	0.094	0.064

*BMA* Broth microdilution assay

^a^Dalbavancin broth microdilution assay was performed according to a recently published study and to CLSI and EUCAST convenients^44,45^. *S. aureus* ATCC 29213 was used as reference strain and yielded a MIC of 0.063 mg/L

**Table 2:** Oligonucleotides used for PCR analysis

Gene	Forward oligonucleotide (5′–3′)	Reverse oligonucleotide (5′–3′)
**Spa** ^a^	TAA AGA CGA TCC TTC GGT GAGC	CAGCAGTAGTGCCGTTTGCTT
**Arc** ^b^	TTG ATT CAC CAG CGC GTA TTG TC	AGG TAT CTG CTT CAA TCA GCG
**Aro** ^b^	ATC GGA AAT CCT ATT TCA CAT TC	GGT GTT GTA TTA ATA ACG ATA TC
**Glpf** ^b^	CTA GGA ACT GCA ATC TTA ATC C	TGG TAA AAT CGC ATG TCC AAT TC
**Gmk** ^b^	ATC GTT TTA TCG GGA CCA TC	TCA TTA ACT ACA ACG TAA TCG TA
**Pta** ^b^	GTT AAA ATC GTA TTA CCT GAA GG	GAC CCT TTT GTT GAA AAG CTT AA
**Tpi** ^b^	TCG TTC ATT CTG AAC GTC GTG AA	TTT GCA CCT TCT AAC AAT TGT AC
**yqil** ^b^	CAG CAT ACA GGA CAC CTA TTG GC	CGT TGA GGA ATC GAT ACT GGA AC

*Spa* Staphylococcal protein A, *arc* carbamate kinase, *aroe* shikimate dehydrogenase, *glpf* glycerol kinase, *gmk* guanylate kinase, *pta* phosphate acetyltransferase, *tpi* triosephosphate isomerase, *yqil* acetyle coenzyme A acetyltransferase

^a^Oligonucleotides for spa typing

^b^Oligonucleotides for MLST

By using a commercial multiplex PCR assay (Genotype-MRSA^®)^, all isolates tested were found to be negative for the presence of *mecA, mecC*, and PVL.

### Auxotrophy testing

For the two SCVs (DR-I3 and DR-I4) no auxotrophic reaction (increased growth around the impregnated filter disks) for factor V, factor X, hemin, menadione, thiamin, thymidine, or tween could be observed at any concentration investigated, after an aerobic or anaerobic incubation of 24–48 h at 36 °C ( ± 1 °C).

### Strain typing

Spa types and MLST of the four *S. aureus* isolates (DR-I1–DR-I4) were t709 and ST 22 [carbamate kinase (arc) 7, shikimate dehydrogenase (aroe) 6, glycerol kinase (glpf) 1, guanylate kinase (gmk) 5, phosphate acetyltransferase (pta) 8, triosephosphate isomerase (tpi) 8, and acetyle coenzyme A acetyltransferase (yqil) 6], respectively. Thus, the four strains were shown to be indistinguishable by spa typing and MLST suggesting at least close relatedness.

### Pulse field gel electrophoresis

The isolates DR-I1, DR-I2, and DR-I3 were found to be indistinguishable with regards to the criteria for interpretation of chromosomal DNA restriction patterns by PFGE, as they showed the same number and apparent size of bands. For strain DR-I4, one additional band was observed ( > 1000 kb). Thus, the isolate was considered to be closely related but not indistinguishable^19^.

### Electron microscopic analysis

The scanning micrographs revealed no major morphologic differences between the three tested isolates (DR-I1, DR-I3, and DR-I4). However, the respective number of bacterial cells in the process of cell division appeared markedly reduced for strain DR-I1 when compared to DR-I3 and DR-I4 (Fig. 2a–f), wherefore this finding was validated by TEM by counting ~200 cells per isolate (Fig. 3a). The relative amounts of bacterial cells in the process of cell division were 35.3% for DR-I1, 66.5% for DR-I3, and 65.7% for DR-I4. Thus, if compared to DR-I1 both SCVs (DR-I3 and DR-I4) demonstrated significantly more bacterial cells during the process of cell division (*p* < 0.0001). Furthermore, a significant difference in cell wall thickness (*p* < 0.0001 for all) was observed between the dalbavancin-susceptible isolate DR-I1 (31 nm, SD ± nm) and the dalbavancin non-susceptible strains DR-I3 (56 nm, SD ± 1 nm) and DR-I4 (64 nm (SD ± 1 nm).

### Growth experiment

Growth rates and doubling times of three *S. aureus* isolates were 0.475 /h (SD ± 0.011) and 1.462 h (SD ± 0.035) for DR-I1, 0.276 /h (SD ± 0.038) and 2.554 h (SD ± 0.322) for DR-I3, and 0.150 /h (SD ± 0.033) and 4.842 h (SD ± 1.068) for DR-I4. Significant differences of growth rates and doubling times were observed comparing DR-I1 to DR-I3 (for both *p* < 0.0001); DR-I1 to DR-I4 (for both *p* < 0.0001) or DR-I3 to DR-I4 (*p* < 0.0001 and *p* = 0.0004), respectively.

### Whole genome sequence analysis

The genetic differences among the four *S. aureus* isolates are shown in Table 3. Briefly, by comparison of DR-I2, DR-I3, and DR-I4 to DR-I1, eight sequence variants were observed. Five of these mutations were single nucleotide polymorphisms (SNPs) in *pbpB*, *rpoB*, *stp1, fusA*, and an unknown gene of a hypothetical protein. Additionally, a 534 bp deletion in the DHH domain of GdpP, a 10.509 bp deletion, involving at least six protein encoding genes (CAAX amino protease, DNA primase, integrase, pathogenicity island protein, phage capsid protein, permease, XRE family transcriptional regulator), and a nucleotide transition through a transposon in the 30S ribosomal protein S16 were found.

**Table 3:** Genetic differences among the four consecutively obtained isolates

Mutation (nucleotide no. in DR-I1)	Locus^a^	Gene name	Gene function	Effect of mutation	Presentation			
					DR-I1	DR-I2	DR-I3	DR-I4
G to A (136230)	jcf180000001136	*pbp2*	penicillin binding protein	G146R	−	−	+	+
T to G (26436)	Jcf7180000001176	*rpoB*	DNA-directed RNA polymerase subunit beta	R436H	−	−	+	+
Deletion A (112755)	jcf7180000001168	*stp1*	serine/threonine protein kinase Stk1	frameshift	−	+	+	+
Insertion A (30413)	jcf7180000001177		hypothetical protein SA_21	frameshift	−	−	+	+
Deletion (40976–40440)	jcf7180000001177		DHH domain of GdpP^b^	534 bp frameshift	−	−	+	+
Deletion (207587–218096)	jcf7180000001135		permease WP_061734446.1	10.509 bp	−	−	+	+
			CAAX amino protease WP_000801979.1		−	−	+	+
			pathogenicity island protein AKJ49778.1		−	−	+	+
			phage capsid protein WP_085065008.1		−	−	+	+
			DNA primase WP_031905599.1		−	−	+	+
			XRE family transcriptional regulator WP_000583241.1		−	−	+	+
			integrase BAM28662.1		−	−	+	+
C to T (33629)	jcf7180000001176	*fusA*	translation elongation factor G	H475Y	+	−	−	−
CAAAAGAAAGCTAAG to TGTAAATGGCGGTTA (90560)	jcf7180000001168	16S rRNA	modified 3′-end of 30S ribosomal protein S16	Altered 3′ end	−	−	+	+

^a^Gene identification number in the first susceptible isolate DR-I1

^b^GGDEF domain protein containing phosphodiesterase

## Discussion

Up to now, emergence of in vivo induced dalbavancin non-susceptibility was only described in two case reports. One case presented a 27-year-old female with tricuspid valve infective endocarditis who consecutively received vancomycin, daptomycin, and dalbavancin^20^. An increase of vancomycin- and daptomycin MICs, however, was already observed before starting with dalbavancin and no lipoglycopeptide MICs were determined at this time. The second case, reported by Werth et al., described a MRSA central line-associated bloodstream infection treated with vancomycin followed by a single dose of dalbavancin^8^. Two weeks after administration of dalbavancin, urine cultures revealed growth of MRSA exhibiting vancomycin-intermediate- and dalbavancin non-susceptibility (MIC 0.5 mg/L). In that study, whole genome analysis of the urine isolate yielded mutations in *ompR, llm, mgtE*, and *yvqF*, whereas *yvqF* variants have previously been implicated in glycopeptide resistance pathways^8–15^. Thus, based on the close relatedness of dalbavancin to vancomycin and the previously reported emergence of glycopeptide resistance under treatment with daptomycin, these findings might at least partially be attributed to the antimicrobial pretreatment in these cases^21^.

**Fig. 1:**
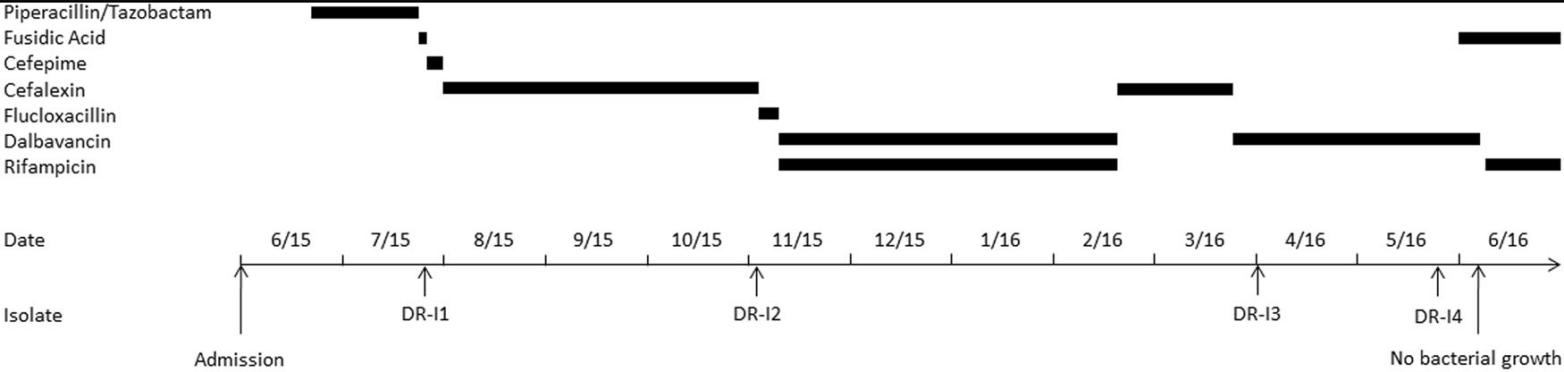
**Time history of antimicrobial usage and bacterial isolation**

In the present study we consecutively isolated dalbavancin non-susceptible and teicoplanin-resistant *S. aureus* SCVs which emerged during long-term dalbavancin therapy of a cardiac device-related endocarditis, without glycopeptide or lipopeptide pretreatment (Fig. 1).

One potential explanation for the phenotypic differences regarding dalbavancin susceptibility is that the obtained isolates were distinct and unrelated *S. aureus* strains. This, however, is highly unlikely as all four strains were determined to be indistinguishable or at least closely related by standard typing methods (*spa*, MLST, and PFGE), and WGS revealed a small number of only eight sequence variants between the four isolates.

Out of these eight sequence variants, *pbp2* and the DHH domain of GdpP were identified as the most probable candidates for the observed glycopeptide/lipoglycopeptide non-susceptible phenotype in the present study. For the penicillin binding protein encoded by the gene *pbp2*, a SNP led to a significant amino acid substitution from the non-polar amino acid glycine to the basic amino acid arginine, which might have influenced the protein structure and thus its activity. Although, this specific mutation has not been previously described in the literature, the overexpression of *pbp2* has been implicated in vancomycin-intermediate *S. aureus* (VISA) phenotype by several studies, demonstrating smaller colony size, lower growth rates, thicker cell walls, decreased lysostaphin susceptibility, and decreased beta-hemolysis^22–25^. Furthermore, the 534 bp deletion in the DHH domain of the GGDEF domain protein containing phosphodiesterase (GdpP) may contribute to the observed phenotypic alterations by impairment of enzyme activity. As described by Corrigan et al., this might have led to elevated c-di-AMP levels and, thus, to an increased amount of cross-linked peptidoglycan^26^. The single nucleotide substitution in the *rpoB* gene, which encodes the β-subunit of RNA polymerase, led to an amino acid change from arginine to histidine, both basic amino-acids wherefore structural and/or functional alterations are unlikely. The variant in the serine/threonine kinase encoded by *stp1* was not only observed in the isolates DR-I3 and DR-I4, demonstrating increased glycopeptide/lipoglycopeptide MICs, but also in the susceptible isolate DR-I2. Thus, although there are some data indicating the involvement of *rpoB* and *stp1* in the VISA phenotype of *S. aureus* isolates, both SNPs are highly unlikely to contribute to the dalbavancin non-susceptible phenotype shown in the present study^12,27^. With regard to the high level resistance against fusidic acid observed in the first isolate (DR-I1), a SNP in the elongation factor G encoding gene *fusA* was previously described to result in an amino acid change from the basic amino acid histidine to the polar amino acid tyrosine^28^. The significance of mutations observed in CAAX amino protease, DNA primase, a hypothetical protein, 30S ribosomal protein S16, integrase, pathogenicity island protein, permease, phage capsid protein, and XRE family transcriptional regulator, if there is any, remains unclear. However, none of these variants were described as contributing factors to glycopeptide/lipoglycopeptide resistance mechanisms of *S. aureus*.

**Fig. 2:**
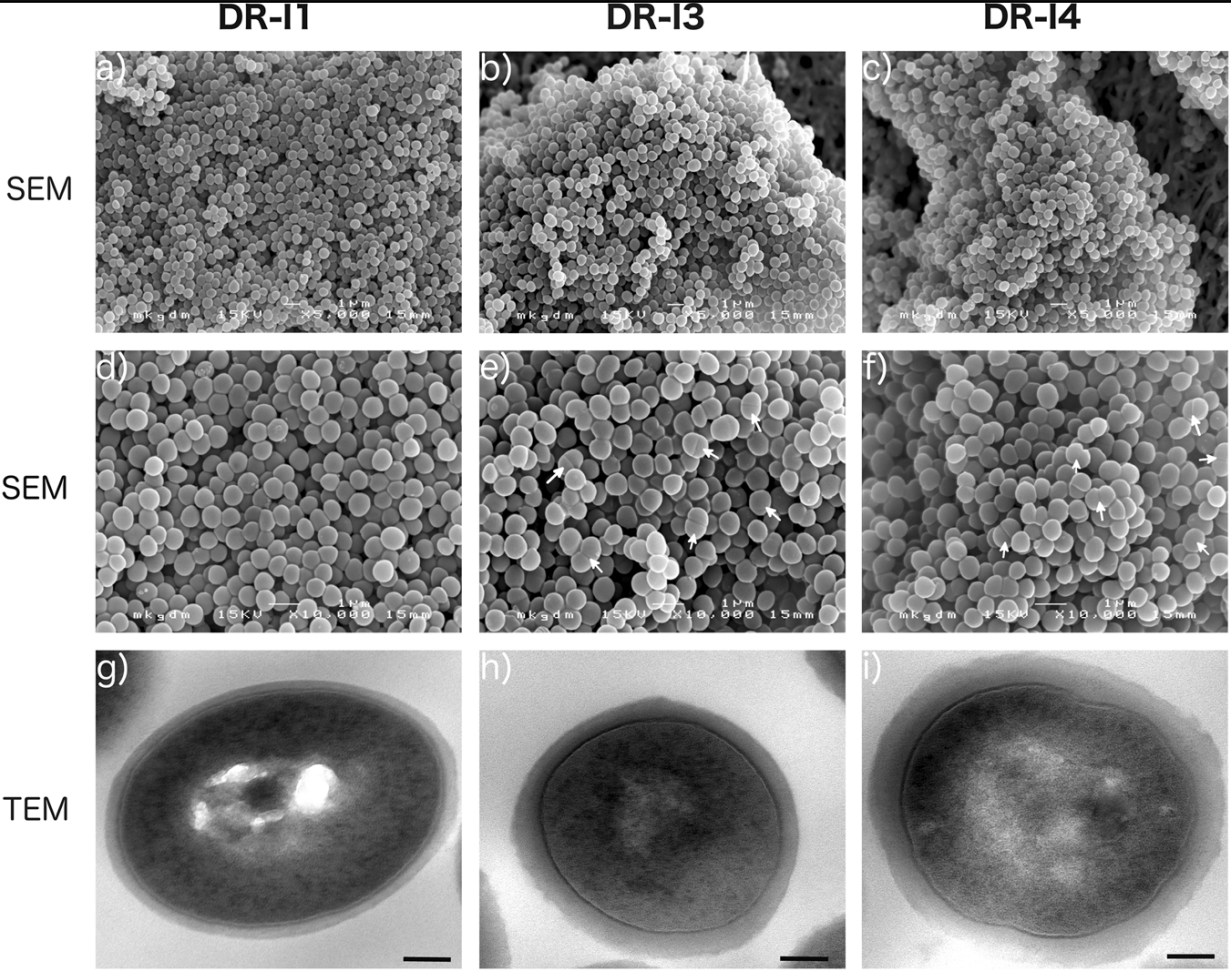
Ultrastructural analysis of dalbavancin non-susceptibility. Scanning electron micrographs at a magnification of ×5000 (**a**, **b**, **c**) and ×10,000 (**d**, **e**, **f**), as well as transmission electron micrographs at a magnification of ×30,000 (**g**, **h**, **i**). White arrows indicate dividing bacterial cells

**Fig. 3:**
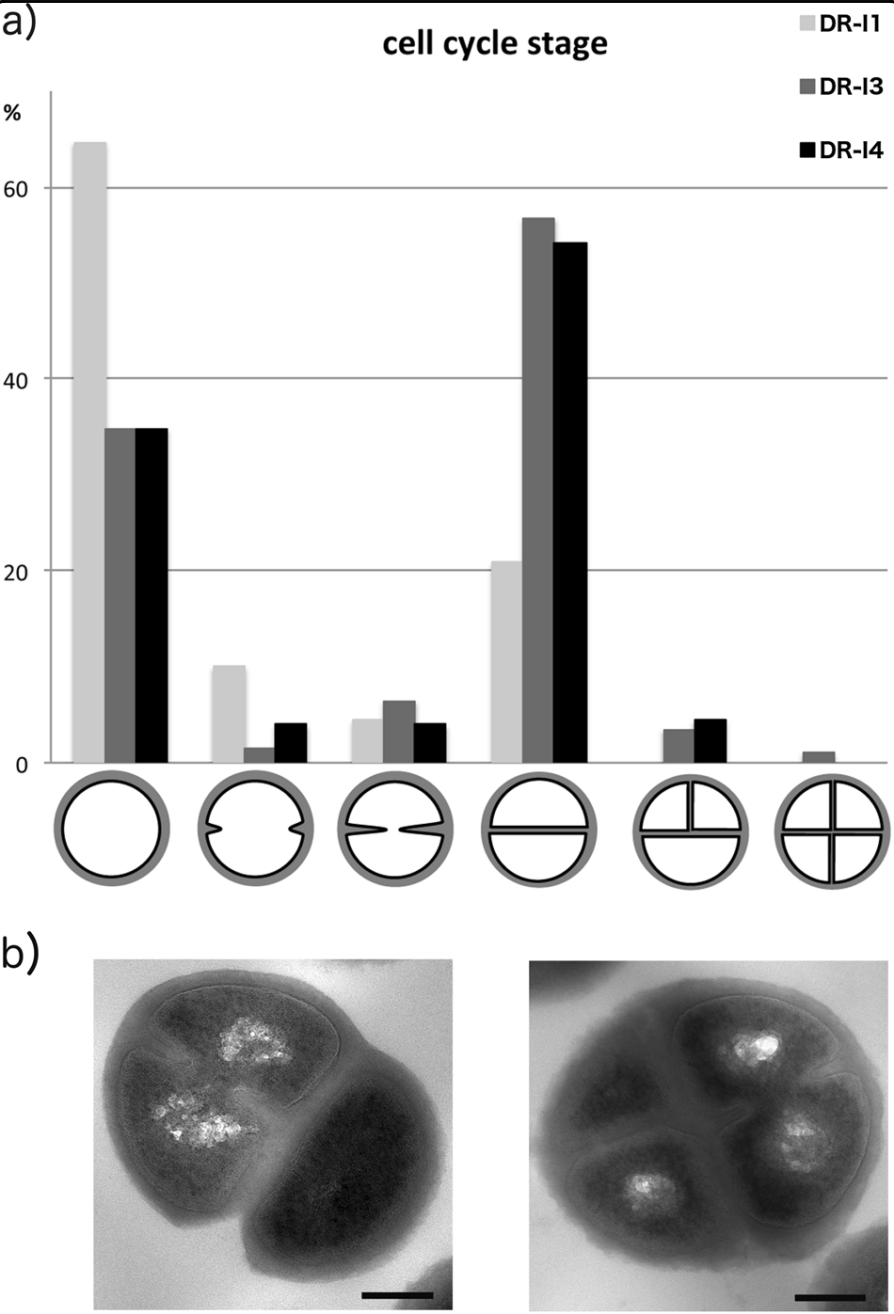
Relative numbers of bacterial cells in specific cell cycle stages. **a** Relative numbers of bacterial cells per morphologic cell cycle stage presented as % of 200 counted cells. **b** Exemplary pictures of irregular cell division observed in the two isolates DR-I3 and DR-I4

In summary, we isolated two dalbavancin non-susceptible and teicoplanin resistant pinpoint SCVs whereof the last isolate (DR-I4) demonstrated a 16- and 84-fold increase in MICs, respectively (Table 1). Compared to the dalbavancin-susceptible isolate DR-I1, the ultrastructural analysis by SEM and TEM revealed a consecutive increase in cell wall thickness (1.8-fold in DR-I3 and 2.1-fold in DR-I4) (Fig. 2g–i) as well as incomplete or multiple cross walls consistent with impaired cell separation (Fig. 3a, b) resulting in significantly reduced growth rates.

Altogether, similar phenotypic changes have been previously described in the literature in electron transport deficient SCVs, VISA, or daptomycin resistant *S. aureus* strains but strikingly the isolates obtained in the present study were neither vancomycin/daptomycin resistant nor demonstrated an auxotrophy or an already known mutation. However, for teicoplanin, the most closely related antimicrobial to dalbavancin, susceptibility testing revealed a highly resistant phenotype strongly implicating a novel resistance mechanism against dalbavancin and teicoplanin. Nevertheless, prior antibiotic courses, particularly with cell wall active drugs like β-lactams, might have selected for organisms more likely to become SCVs and should therefore be taken into account during such extraordinary clinical courses as described in the present study.

Of interest, as described previously, dalbavancin MIC ratios (Etest-MIC/broth microdilution-MIC) were 0.75 (DR-I1 and DR-I2) and 0.38 (DR-I3 and DR-I4), demonstrating markedly lower MICs obtained by Etests when compared to the reference broth microdilution method^29^. Further, in contrast to the study by Jones et al., who investigated the potential use of vancomycin MICs as a surrogate parameter of dalbavancin susceptibility, dalbavancin, and vancomycin MICs were demonstrated to be notably diverging in this study^30,31^. Thus, with regards to the distinct phenotype observed in the present study, not only screening for dalbavancin resistance, but also the interpretation of MICs and therefore treatment decisions in clinical routine might be challenging.

One potential explanation for the in vivo induction of dalbavancin non-susceptibility and teicoplanin resistance, described in the present study, is the extensive terminal half-life of dalbavancin paired with the treatment transition to cefalexin (February/March 2016)^5–7^. Additionally, despite the fact that SCVs, per se, have not been established to be the cause of treatment failure they might be a contributing factor for biofilm formation on foreign bodies such as the PM-wire of the patient, leading to markedly increased antibiotic resistance due to several factors including glycocalyx mediated limitation of drug diffusion, increased expression of efflux pumps, accumulation of exoenzymes, and reduced metabolic activity^32–34^. Thus, the low trough levels of dalbavancin in conjunction with the markedly higher antimicrobial concentrations needed for eradication of biofilms compared to the eradication of planktonic cells (10- to 1000-fold) might have led to a prolonged overlap with the mutant selection window highlighting the need for sufficient source control to achieve clinical cure in case of biofilm mediated infections such as cardiac device-related endocarditis^35–38^.

In conclusion, we isolated vancomycin-susceptible and dalbavancin non-susceptible /teicoplanin-resistant *S. aureus* SCVs in a patient with a cardiac device-related infective endocarditis during the currently longest reported duration of dalbavancin treatment of approximately thirty weeks. The bacterial phenotype demonstrated a stepwise alteration regarding cell wall thickness, impairment of cell separation, growth rates and antimicrobial susceptibility patterns. These changes were associated with eight, previously not described, sequence variants of which the mutations in *pbp2* and the DHH domain of GdpP were the most probable candidates.

Dalbavancin might be a feasible alternative for long-term OPAT of difficult to treat infections. However, its extensive half-life might be a double-edged sword with an increased risk to induce resistances particularly in cases of biofilm formation and insufficient source control^38^.

## Materials and methods

### Ethical approval

Ethical approval for this study was waived by the local ethics committee and an informed consent was obtained from the patient prior to all study related activities.

### Patient characteristics and antimicrobial usage

We report on a 36-year-old male who was transferred to the Department of Infectious Disease and Tropical Medicine at the Medical University of Vienna in June 2015 with suspected cardiac device-related endocarditis after Senning-surgery (1980) and pacemaker (PM) implantation (1985) due to transposition of the great arteries and atrial fibrillation. The patient was receiving intravenous antimicrobial therapy (Fig. 1) and presented with an inflamed PM entry, fever (up to 39.6 °C), chills and night sweats. Repeated transesophageal echocardiographies were performed but no radiological evidence for infective endocarditis was found. Blood cultures revealed *Staphylococcus aureus*, sensitive to all routinely tested antimicrobials. After two unsuccessful efforts of PM explantation and transition of the antimicrobial therapy to p.o. fusidic acid, a bacteraemic relapse occurred (strain no. DR-I1). After susceptibility patterns were received, antibiotic therapy was transitioned to p.o. cefalexin over a period of three months (Fig. 1). One day after the end of treatment, the patient again experienced a bacteraemic relapse and presented at the emergency department (strain no. DR-I2). On patient’s request surgical explantation of the remained PM-wire was postponed to April 2016 and, thus, the initially started treatment with flucloxacillin was transitioned to an OPAT with dalbavancin (plus rifampicin p.o.; Fig. 1). Microbiological examination of the explanted PM-wire revealed a methicillin-resistant *S. aureus* (MRSA) SCV (strain no. DR-I3) demonstrating a slightly increased minimal inhibitory concentration (MIC) for vancomycin (2 mg/L). Despite the ongoing OPAT with dalbavancin, the patient presented again to the outpatient department at the end of May when a dalbavancin non-susceptible MRSA SCV (strain no. DR-I4) was detected in multiple sets of blood cultures. Thus, according to the antimicrobial susceptibility profile, treatment was transitioned to p.o. fusidic acid plus rifampicin resulting in negative blood cultures and improved clinical conditions of the patient (Fig. 1).

### Strain typing

To assess the relatedness of the four isolates spa typing, by amplification and sequencing of the staphylococcal protein A (*spa*) gene X region, as well as multi locus sequence typing (MLST) were performed as previously described^39–41^.

The amplicons were sequenced using the genetic analyzer 3130 (Applied Biosystems Inc, Foster City, California, USA). Obtained sequences were translated to spa types using the Ridom SpaServer database (http://www.spaserver.ridom.de) and MLST was conducted as described by Enright et al. using the accessible database at http://saureus.mlst.net/^41,42^.

### Pulse field gel electrophoresis

Pulse field gel electrophoresis (PFGE) was performed as described recently^43^. Briefly, bacterial- and lysostaphin-concentrations had to be increased for the isolates DR-I3 and DR-I4, due to considerably less DNA concentrations which were achieved by using standard protocol. DNA was digested with SmaI, separated using the Rotaphor^®^ (Biometra, Göttingen, Germany) PFGE system and gels were stained with Gelred™ (GelRed™ Nucleic Acid Gel Stain 10,000 × , Biotium Inc., Fremont, California, USA). Isolates were considered to be indistinguishable if their restriction patterns showed the same number and apparent size of bands. Furthermore, isolates were defined as closely related, possibly related or different if 1–3, 4–6, or ≥7 fragment differences occurred, respectively^19^.

### Antimicrobial susceptibility testing

MICs of each strain were determined for cefoxitin, ciprofloxacin, clindamycin, dalbavancin, daptomycin, doxycycline, fosfomycin, fusidic acid, gentamicin, linezolid, oxacillin, rifampicin, teicoplanin, trimethoprim/sulfamethoxazole, and vancomycin using Etests (Liofilchem SRL, Italy) on Mueller-Hinton agar (MHA) plates. According to the recommendations of the Clinical and Laboratory Standards Institute (CLSI) MICs were assessed after incubation for 18–24 h at 36 °C ( ± 1 °C)^44,45^. To validate obtained MICs for dalbavancin, a previously described broth microdilution (BMD) assay in CA-MHB plus polysorbate 80 was performed additionally^44^. Obtained susceptibility data were interpreted according to the most recent clinical breakpoints of the European Committee on Antimicrobial Susceptibility Testing (EUCAST)^46^.

To test all isolates for the presence of Panton Valentine leukocidin (PVL) and methicillin-resistance genes (*mecA* and *mecC*), the Genotype-MRSA^®^ test (Hain Lifescience GmbH, Nehren, Germany), which is based on DNA-STRIP technology, was used.

### Auxotrophy testing

For auxotrophy testing, MHA plates were inoculated with 10^4^, 10^6^ and 10^8^ colony forming units (CFU) of the isolates DR-I3 and DR-I4. Six millimeter filter disks (Macherey-Nagel GmbH & Co. KG, Düren, Germany) were impregnated with tween (0.1%, 1 and 10%) as well as hemin, menadione, thymidine and thiamin (all from Sigma–Aldrich, St Louis, Missouri, USA) at concentrations of 1 µg/mL, 10 µg/mL, 100 µg/mL, 1000 µg/mL, 2000 µg/mL, and 5000 µg/mL. In addition, commercially available hemin and NAD + disks (factor X and factor V disks, Mast Group Ltd., Bootle, United Kingdom) were used. Isolates were considered auxotrophic if a zone of increased growth could be observed around the impregnated filter disks after an aerobic, as well as an anaerobic incubation for 24 h at 36 °C ( ± 1 °C)^47,48^.

### Growth experiment

Growth curves were performed by OD600 measurement, using a Tecan Magellan Sunrise Spectrophotometer^49^. Bacterial cultures were grown overnight in Lennox broth (LB) to obtain saturated suspensions. The final inoculum was obtained by 1:1000 dilution of the bacterial suspension in fresh LB medium and inoculated in flat-bottomed 96 well-plates. Measurements were taken at 10-minute intervals for 20 h at 28 °C. The experiments were performed as independent triplicates using four wells per strain and fresh LB medium was used as negative control each time. Growth rates and doubling times were determined by using the non-linear least-squares Levenberg-Marquardt algorithm which is implemented in the Growthcurver R package by Sprouffske and Wagner^50^.

### Electron microscopy

All isolates were incubated overnight on MHA plates at 36 °C ( ± 1 °C) and re-suspended in 0.1 M phosphate buffer (pH 7.2). Cell pellets were obtained by centrifugation (10,000× *g* for 10 min at 4 °C) and fixed with 2.5% glutaraldehyde in 0.1 M phosphate buffer for 20 min for scanning electron microscopy (SEM) or 24 h for transmission electron microscopy (TEM). For SEM, samples were dehydrated in ascending ethanol series and re-suspended in hexamethyldisilazane (HMDS). After complete evaporation of HMDS samples were sputter-coated with gold (Sputter Coater, SC 502, Polaron, Fisons Instruments^®^, England) and examined using a scanning electron microscope (JSM 6310, Jeol Ltd.^®^, Japan). Images were taken at an acceleration voltage of 15 kV^51^.

TEM analysis was performed as described previously^52^. Briefly, samples were incubated in 1% OsO_4_ and embedded in Epon resin. 70 nm ultra-thin sections were imaged with an FEI Tecnai20 electron microscope equipped with a 4 K Eagle-CCD camera. Images were processed with Adobe Photoshop. For measurements of the thickness of the cell walls, ~100 non-dividing cells each were imaged. The ImageJ software package was used for measurements. Cell cycle stages of the three isolates (DR-I1, DR-I3 and DR-I4) were assessed by counting approximately 200 cells each.

### Whole genome sequence analysis

Samples were prepared and sequenced employing Nextera XT DNA Sample Library Preparation Kit, Nextera XT Index Kit and Miseq Reagent Kit v3 (Illumina, San Diego, USA). Whole genome sequencing (WGS) was performed on all four isolates (DR-I1 – DR-I4) using an Illumina MiSeq system (Illumina, San Diego, USA) with a 300 bp paired-end library, resulting in 1442240, 1760908, 2112322, and 1303370 past filter reads (~156, 190, 280, and 141-fold genome coverage), respectively. The reads were trimmed and filtered with trimmomatic-0.33^53^ and de novo assembled by means of MaSuRCA-3.2.4^54^. The resulting contigs were compared against each other by means of mauve version 20150226 build 10 ^55^ and the resulting gaps and SNPs were verified/discarded by means of harvester [in press] and UGENE^56^.

### Statistics

Results were analyzed and plotted using GraphPad Prism version 6. For comparison of mean growth rates, doubling times and cell wall thickness ANOVA was used as global test and unpaired *t*-tests with Welch’s correction as post hoc analysis. For comparison of the relative amounts of bacterial cells in the process of cell division a Chi-square test was used.

## Data Availability

WGS data for the four genomes have been deposited in the NCBI genome database (BioProject SRP158128).
